# Seroepidemiology of Enterovirus D68 in Xiamen Children: A Cross-Sectional Study in 2022

**DOI:** 10.3390/v18080903

**Published:** 2026-08-17

**Authors:** Liting Wang, Qiguo Zhu, Yi Lu, Yuanyuan Wu, Zhifeng Ke, Qingbing Zheng, Longfa Xu, Tong Cheng, Rui Zhu, Jun Shen

**Affiliations:** 1 Infectious Disease Department, Children’s Hospital of Fudan University, National Children’s Medical Center, Shanghai 201102, China; 24211240020@m.fudan.edu.cn; 2 Xiamen Children’s Hospital, Xiamen Branch of Children’s Hospital of Fudan University, Xiamen 361006, China; zhuqiguo2007@163.com (Q.Z.); papamaker@126.com (Y.L.); 3 State Key Laboratory of Vaccines for Infectious Diseases, Xiang An Biomedicine Laboratory, School of Life Sciences, School of Public Health, Xiamen University, Xiamen 361005, China; 21620220157001@stu.xmu.edu.cn (Y.W.); 21620241153524@stu.xmu.edu.cn (Z.K.); abing0811@xmu.edu.cn (Q.Z.); longfaxu@xmu.edu.cn (L.X.); tcheng@xmu.edu.cn (T.C.); 4 Pediatric Department, Children’s Hospital of Fudan University at Qidong, Nantong 226200, China

**Keywords:** enterovirus D68, seroprevalence, neutralizing antibodies, children, seroepidemiology

## Abstract

Enterovirus D68 (EV-D68) is a re-emerging pathogen associated with severe acute flaccid myelitis (AFM) and pneumonia, predominantly in children but also capable of causing severe disease in immunocompromised adults. Seroprevalence data are essential for understanding population immunity, age-specific susceptibility, and viral transmission dynamics; yet, such data for the period after the COVID-19 pandemic are scarce. We conducted a cross-sectional serological survey to characterize the neutralizing antibody profile of EV-D68 among children in Xiamen, China, in 2022. A total of 453 children aged 0–16 years (217 with acute respiratory tract infection [ARTI] and 236 without) were enrolled. Neutralizing antibody (NtAb) titers against the EV-D68 STL strain were measured using a microneutralization assay, seropositivity was defined as a titer ≥1:16. Multivariable regression and analysis of covariance were used to adjust for age and sex. Overall seroprevalence was 96.0% (435/453), exceeding 90.0% in every age subgroup (0–1 y: 100%; 1–3 y: 97.6%; 3–5 y: 92.6%; ≥5 y: 96.2%). The geometric mean titer (GMT) was 76.78 (95% CI: 68.32–85.24). After adjustment, ARTI status was not significantly associated with seropositivity (adjusted OR = 1.42, 95% CI: 0.46–4.41, *p* = 0.542). In an exploratory comparison, children with ARTI showed nominally higher antibody titers than those without (Mann–Whitney *U* test, *p* = 0.003), although the absence of EV-D68-specific PCR testing precludes etiologic attribution of respiratory symptoms to EV-D68 infection. Infants < 1 year showed the highest GMT (86.31), while children aged 1–3 years had the lowest GMT (63.52) and the largest low-titer fraction, indicating a susceptibility gap. Our study revealed EV-D68 circulated endemically in this pediatric population, establishing a high population immune baseline (>90.0%) with distinct age-dependent patterns. These findings provide key reference data for ongoing serosurveillance, outbreak risk assessment, and the evaluation of future vaccines or immunoprophylactic strategies should they become available.

## 1. Introduction

EV-D68 is a non-polio enterovirus of the species *Enterovirus D* within the family *Picornaviridae* that primarily causes respiratory illness but has garnered global public health attention due to its strong association with AFM—a severe neurologic complication predominantly affecting children [1,2]. First identified in 1962 from four children with bronchiolitis and pneumonia in California, EV-D68 remained a sporadic pathogen until 2014, when a large outbreak across the United States marked its re-emergence as a major pediatric threat [3,4]. Since then, biennial epidemic peaks have been observed, each accompanied by a surge in AFM cases, highlighting the need for continuous epidemiological surveillance [5,6].

Seroprevalence studies are fundamental to understanding the epidemiology of EV-D68 because they reveal the cumulative burden of infection, age-dependent susceptibility, and the duration and magnitude of humoral immunity at the population level [7]. Such data are indispensable for identifying high-risk age groups, assessing the potential for future outbreaks, and guiding public health surveillance.

In the United States, post-outbreak serosurveys have documented the persistence of neutralizing antibodies in young children, even those born after the 2014 outbreak, suggesting ongoing community transmission [8]. In Europe, studies from the Netherlands and England indicated that by age 2 approximately 50.0% of children have been infected, underscoring the early and intense exposure that occurs in many settings [9,10]. In Asia, a longitudinal serosurvey in Japan spanning 1976–2019 confirmed stable endemic circulation with periodic epidemic boosts, providing a valuable baseline for assessing long-term trends [11], while a study from Malaysia showed similar age-dependent acquisition patterns [12]. At the global level, a recent systematic review by Jorgensen et al. (2025) collated global age-stratified seroprevalence estimates, demonstrating that seropositivity rises rapidly through childhood and approaches 100% by early adulthood, with no evidence of decline thereafter [13]. In China, retrospective surveys from Beijing have shown widespread EV-D68 exposure between 2004 and 2017, with age-specific patterns generally consistent with global observations [14,15,16].

Despite these contributions, the seroepidemiological landscape of EV-D68 in the period following the COVID-19 pandemic remains poorly characterized. The global interruption of biennial circulation during 2020–2021, driven by the widespread implementation of COVID-19 non-pharmaceutical interventions (e.g., social distancing, mask mandates, school closures), followed by resurgence in 2022, raises questions about the current immune landscape and the potential for large outbreaks [17]. Without updated serological baselines, it is difficult to determine whether the interruption of transmission during the COVID-19 pandemic has created an accumulation of susceptible children, or whether endemic circulation persisted at lower levels. Furthermore, contemporary circulating strains have undergone genetic evolution, with subclade B3 becoming predominant and a novel D3 subclade emerging since 2018, potentially affecting transmissibility and antigenic properties [17,18,19]. These changes underscore the need for updated serological baselines to monitor population immunity and detect shifts in age-specific susceptibility that may alter outbreak dynamics.

To address this gap, we conducted a cross-sectional seroprevalence survey among children in Xiamen, China, between May and November 2022—a period after the relaxation of strict public health measures. The primary objective of this study was to estimate age-specific EV-D68 seroprevalence and neutralizing antibody GMTs among children in Xiamen, China, in 2022, and to establish a robust serological baseline for future surveillance. As a secondary, exploratory analysis, we also compared antibody profiles between children presenting with ARTI and those without respiratory symptoms, recognizing that EV-D68-specific molecular testing was not available and that this comparison cannot establish etiologic attribution.

## 2. Materials and Methods

### 2.1. Study Design and Participants

This was a hospital-based cross-sectional serological survey conducted at Xiamen Children’s Hospital between May and November 2022. A total of 453 children aged 0–16 years were enrolled, comprising 217 children with a history of ARTI (outpatient or inpatient) and 236 children without ARTI who underwent routine blood tests for non-infectious reasons (e.g., pre-surgical screening or routine physical examinations). Inclusion criteria: (1) Age 0–16 years; (2) Complete clinical records and available serum samples. Exclusion criteria: (1) Primary or secondary immunodeficiency; (2) Intravenous immunoglobulin therapy within 3 months prior to sampling; (3) Duplicate samples from the same individual (only the sample with the higher titer was retained); (4) Incomplete clinical or laboratory data. ARTI was defined by the treating clinician based on the presence of acute-onset respiratory symptoms (cough, rhinorrhea, sore throat, or dyspnea) with or without fever (tympanic temperature ≥ 38.0 °C), of ≤14 days’ duration, documented in the electronic medical record at the time of presentation. Radiographic confirmation was not required for inclusion. The non-ARTI group comprised children undergoing routine blood draws for non-infectious indications (e.g., pre-surgical screening, health examinations) who had no documented respiratory symptoms or fever within the preceding 14 days. All participants were recruited from the same hospital catchment area in Xiamen, a coastal city in southeastern China with a population of approximately 5.3 million.

### 2.2. Data Collection

Demographic data (age, sex) and clinical information (ARTI diagnosis, confirmed respiratory pathogens if available) were extracted from electronic medical records. For children with ARTI, pathogen testing was performed using multiplex PCR panels at the clinician’s discretion. Of note, EV-D68-specific PCR testing was not systematically performed for ARTI cases, as EV-D68 primers were not included in the routine respiratory pathogen panel at the study site during the study period. Consequently, the etiologic role of EV-D68 in the respiratory symptoms of the ARTI group cannot be confirmed, and the ARTI–non-ARTI comparison is considered exploratory. Respiratory pathogen testing was performed at the treating clinician’s discretion using multiplex PCR panels that targeted common respiratory viruses (influenza A/B, respiratory syncytial virus, adenovirus, rhinovirus, human metapneumovirus, parainfluenza virus types 1–4) and atypical bacteria (*Mycoplasma pneumoniae*), but did not include enterovirus typing or EV-D68-specific primers.

### 2.3. Microneutralization Assay

EV-D68 neutralizing antibody (NtAb) titers were measured using a validated ELISPOT-based microneutralization assay developed at the State Key Laboratory of Vaccines for Infectious Diseases, Xiamen University [20]. The assay in this study was validated against the conventional cytopathic effect (CPE)-based microneutralization assay using a panel of 30 serum samples, showing excellent correlation (Spearman ρ > 0.95) and superior sensitivity for low-titer neutralizing activity.

The EV-D68 reference strain STL (GenBank accession No. KM881710), belonging to clade B and originally isolated during the 2014 US outbreak, was propagated in rhabdomyosarcoma (RD) cells (ATCC, Cat. No. CCL-136, Manassas, VA, USA). RD cells were maintained in Dulbecco’s Modified Eagle Medium (DMEM; Gibco, Cat. No. 11995-065, Waltham, MA, USA) supplemented with 10% heat-inactivated fetal bovine serum (FBS; Gibco, Cat. No. 10091-148, Waltham, MA, USA), 100 U/mL penicillin, and 100 μg/mL streptomycin, and cultured at 37 °C in a 5% CO_2_ atmosphere. For the neutralization assay, RD cells were seeded in 96-well plates at 2 × 10^4^ cells per well and cultured for 24 h to reach approximately 90% confluence.

Serum samples were heat-inactivated at 56 °C for 30 min and serially diluted two-fold from 1:16 to 1:2048 in serum-free DMEM. Each dilution was mixed with an equal volume of virus suspension containing 1.5 × 10^4^ TCID_50_ of EV-D68 STL and incubated at 33 °C for 1 h. The virus–serum mixture was then added to the pre-seeded RD cell monolayers. Each plate included a positive control (hyperimmune rabbit serum raised against purified, formalin-inactivated EV-D68 STL virions; neutralizing titer > 1:2048) and a negative control (cells only, no virus).

After 16 h of incubation at 33 °C in 5% CO_2_, cells were fixed with 0.4% paraformaldehyde in phosphate-buffered saline (PBS) for 45 min at room temperature, permeabilized with 0.1% Triton X-100 in PBS for 10 min, and blocked with 3% bovine serum albumin (BSA) in PBS for 1 h. Infected cells were detected using an in-house EV-D68-specific HRP-conjugated monoclonal antibody 15C5 (diluted 1:5000 in PBS containing 1% BSA), which recognizes a conformational epitope on the VP1 capsid protein with broad reactivity across EV-D68 clades and no cross-reactivity with other enterovirus species (EV-A71, coxsackievirus A16, coxsackievirus B3). Following incubation with 15C5-HRP for 1 h at 37 °C and washing, spots were developed with TMB substrate (Mabtech, Cat. No. 3652-10, Nacka Strand, Sweden) and counted using an automated ELISPOT reader (CTL ImmunoSpot S6 Ultra, Cellular Technology Limited, Shaker Heights, OH, USA).

The neutralization titer was defined as the highest serum dilution achieving >50% reduction in spot counts compared with virus-only control wells (eight replicates per plate). A titer ≥ 1:16 was considered seropositive, consistent with the threshold used in previous international EV-D68 serosurveys. All samples were tested in duplicate, and the geometric mean of the two replicates was used for analysis.

### 2.4. Statistical Analysis

Continuous variables were expressed as median (interquartile range, IQR) for non-normally distributed data and as mean ± standard deviation (SD) for normally distributed data. Categorical variables were presented as frequencies and percentages. Group comparisons were performed using Pearson’s *χ*^2^ test for categorical variables and the Mann–Whitney *U* test or independent *t*-test for continuous variables, as appropriate. Given the significant age difference between ARTI and non-ARTI groups, we employed multivariable logistic regression to identify independent factors associated with EV-D68 seropositivity, adjusting for age group (categorical: 0–1, 1–3, 3–5, ≥5 years), sex, and ARTI status. To evaluate the independent effect of ARTI status on antibody titers (log_2_-transformed), we performed an analysis of covariance (ANCOVA) with age group and sex as covariates. To verify the robustness of the parametric results given the skewed titer distribution, we additionally performed nonparametric sensitivity analyses: the Mann–Whitney U test for pairwise comparison of log_2_-transformed NtAb titers between ARTI and non-ARTI groups, the Kruskal–Wallis test for comparisons across age groups (with Bonferroni-corrected pairwise post hoc tests), and Spearman’s rank correlation to assess the relationship between age (continuous) and log_2_-transformed titers. The Shapiro–Wilk test was used to formally assess the normality of the log_2_-transformed titer distribution.

All analyses were performed using R version 4.2.1 (R Foundation for Statistical Computing, Vienna, Austria). A two-sided *p* < 0.05 was considered statistically significant. To account for multiplicity, we applied the Bonferroni correction to the five individual titer stratum comparisons (adjusted α = 0.05/5 = 0.010) and to the six pairwise Mann–Whitney U comparisons between age groups. The Benjamini–Hochberg procedure was applied to control the false discovery rate for the five predictors in the Firth logistic regression model. Adjusted *p* values are reported alongside nominal *p* values where applicable. Results not surviving correction are explicitly noted as exploratory. Key covariates including daycare attendance, household size (number of children), and recent respiratory virus exposure history were not systematically documented in the electronic medical records at Xiamen Children’s Hospital during the study period and thus could not be incorporated into the regression models.

### 2.5. Ethics Statement

This study was conducted in accordance with the Declaration of Helsinki. Ethical approval was obtained from the Ethics Committee of Xiamen Children’s Hospital (approval No. 2023-129). Written informed consent was obtained from the legal guardians of all participants after full explanation of the study objectives.

## 3. Results

### 3.1. Demographic Characteristics

A total of 453 children were included, with 278 boys (61.4%) and 175 girls (38.6%). The overall median age was 4.0 years (IQR: 2.0–7.0). Children with ARTI were significantly younger than those without ARTI (median 3.0 vs. 5.0 years, *p* < 0.001) and had a lower proportion of children aged ≥ 5 years (34.1% vs. 57.2%, *p* < 0.001) (Table 1). Among the 217 ARTI children, 121 (55.8%) had at least one respiratory pathogen identified, with *Mycoplasma pneumoniae* (27 cases), rhinovirus (26), *Haemophilus influenzae* (15), metapneumovirus (12), and parainfluenza virus (12) being the most common.

### 3.2. Overall EV-D68 Neutralizing Antibody Distribution

The overall seroprevalence was 96.0% (435/453). The distribution of NtAb titers across all participants is shown in Table 2. The majority of children (82.1%) had titers between 1:16 and 1:256, reflecting a predominantly moderate-level humoral immune response.

In univariable stratum-by-stratum comparisons, a nominally higher proportion of low-titer (1:16–1:32) antibodies was observed among non-ARTI children (39.0% vs. 28.6%, nominal *p* = 0.025). However, after Bonferroni correction for the five titer strata tested (adjusted α = 0.010), this difference did not reach statistical significance (adjusted *p* = 0.125). The global χ^2^ test for the overall titer distribution also did not demonstrate a significant difference between groups (χ^2^ = 9.357, df = 4, *p* = 0.053). Given the multiple comparisons performed and the borderline nature of this result, these findings should be regarded as exploratory and interpreted with caution.

### 3.3. Age-Specific Antibody Profiles

**Figure 1:**
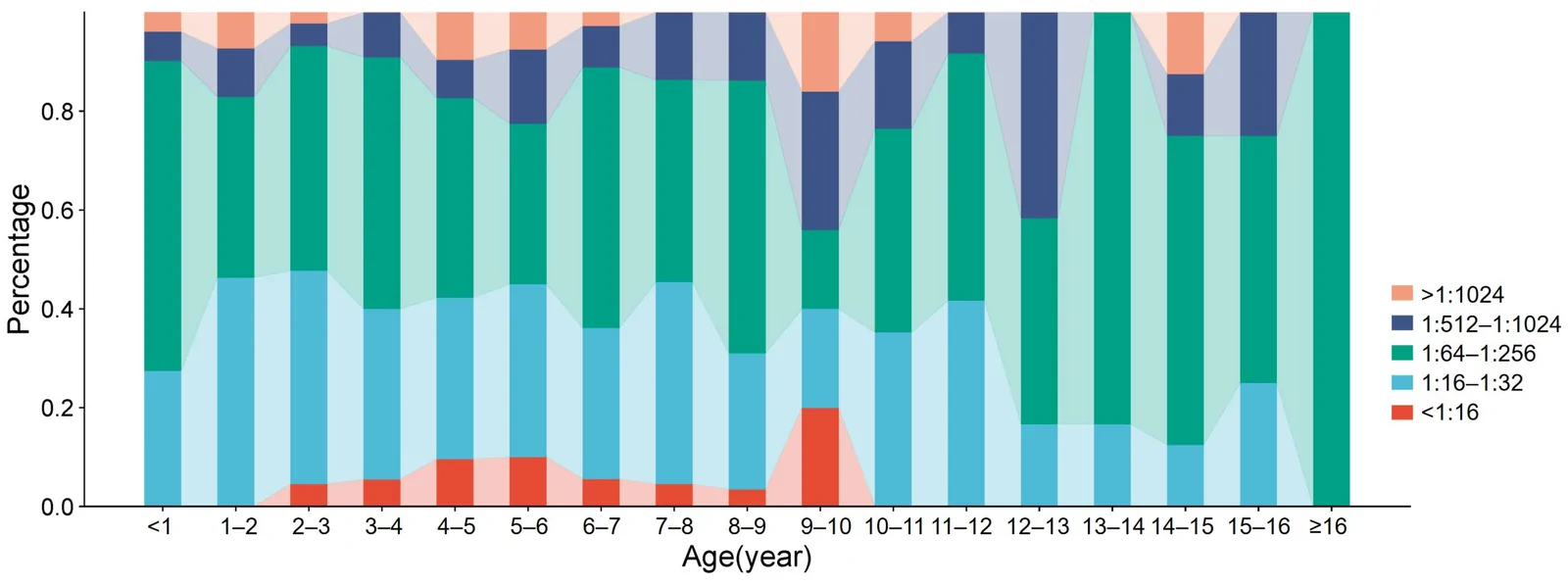
Distribution of EV-D68 neutralizing antibody titers by single year of age. Each vertical bar represents one age year and is subdivided into five titer strata: <1:16 (red), 1:16–1:32 (light blue), 1:64–1:256 (green), 1:512–1:1024 (dark blue), and >1:1024 (orange). The height of each colored segment is proportional to the percentage of children in that age–titer subgroup, with bars summing to 100% for each age.

A linear stacked bar chart visualizing the single-year age and titer distribution is presented in Figure 1. It can be observed that starting from age 2, the positive rate of EV-D68 neutralizing antibodies begins to decline, reaching its lowest point between ages 9 and 10; conversely, the positive rate of neutralizing antibodies remains at 100% both before age 2 and after age 10.

**Figure 2:**
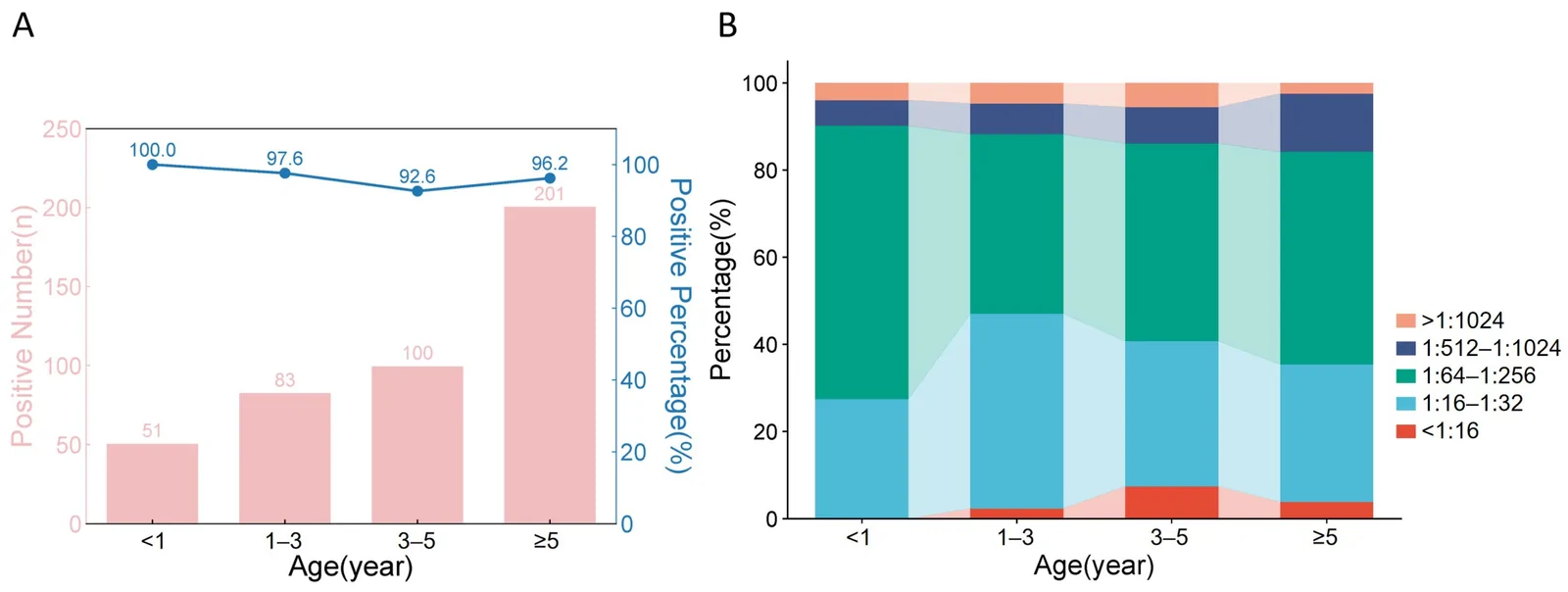
Age-stratified EV-D68: Graphs of the neutralizing antibody positivity rate and titer distribution. (**A**) Neutralizing antibody positivity status among 453 pediatric patients across the <1, 1–3, 3–5, and ≥5-year age groups: The X-axis represents these four age groups; the Y-axis represents the number of positive cases (pink bars indicate the number of positive cases in each corresponding age group, with the left Y-axis showing the scale values for the number of positive cases) and the positivity rate (blue scatter points represent the positivity rate for each corresponding age group, with lines connecting the scatter points illustrating the trend between age groups, and the right Y-axis showing the scale values for the positivity rate). (**B**) Distribution of neutralizing antibody titers among 453 pediatric patients across the <1, 1–3, 3–5, and ≥5-year age groups: The X-axis represents the four age groups; the Y-axis represents the proportion of patients within each age group falling into specific neutralizing antibody titer ranges (<1: 1:16 [red]; 1:16–1:32 [light blue]; 1:64–1:256 [green]; 1:512–1:1024 [dark blue]; >1: 1:1024 [orange]), with the sum of all proportions equaling 100%. (The filling lines between columns represent the trend in the proportion of neutralizing antibody titer ranges across different age groups).

Seroprevalence exceeded 90.0% across all predefined age groups: 100% (0–1 year), 97.6% (1–3 years), 92.6% (3–5 years), and 96.2% (≥5 years), with the lowest seropositivity observed in the 3–5-year age bracket (Figure 2A). The Cochran–Armitage test for trend showed no significant linear trend in seroprevalence across age groups (*p* for trend = 0.152), likely due to the near-saturation of seropositivity.

As shown in Figure 2B, the two neutralizing antibody titer ranges—1:16–1:32 and 1:64–1:256—account for the highest proportions across all age groups, with the 1:64–1:256 range being predominant, particularly evident among children aged 0–1 years. Additionally, cases with extremely high neutralizing antibody titers (1:512–1:1024 and >1:1024) or negative results (<1:16) represent relatively low proportions across all age groups; specifically, the proportion of negative results (<1:16) is zero among children under 1 year of age, while the proportion of neutralizing antibody titers > 1:1024 is the lowest among children aged ≥ 5 years.

Single-year age analysis revealed distinct patterns: infants aged <1 year had the highest GMT (86.31) and the highest proportion of mid-to-high titer antibodies (≥1:64: 72.5%), while children aged 1–3 years showed the lowest proportion of ≥1:64 neutralizing antibodies (52.9%). School-aged children (≥5 years) exhibited stable mid-range titers with a ≥1:64 seropositive proportion of 64.1%. The overall GMT of NtAb among all 453 children was 76.78 (95% CI: 68.32–85.24). Age-specific GMTs were: 86.31 (0–1 y), 63.52 (1–3 y), 69.14 (3–5 y), and 80.47 (≥5 y).

### 3.4. Age- and ARTI-Stratified Antibody Distribution

Table 3 presents a simplified summary of age- and ARTI-stratified seroprevalence and key titer distribution metrics. Within each age stratum, seroprevalence did not differ significantly between ARTI and non-ARTI children (all *p* > 0.05). However, non-ARTI children consistently carried a larger proportion of low-titer (1:16–1:32) sera across most age groups, whereas ARTI children tended to have higher proportions of elevated titers (≥1:512), particularly among infants < 1 year. Notably, all 18 infants in the non-ARTI group were seropositive, yet none reached titers ≥ 1:512, while among the 33 ARTI infants, 9.1% had titers ≥ 1:512.

### 3.5. Factors Associated with Seropositivity

Given the small number of seronegative children (*n* = 18), we performed Firth’s penalized logistic regression to identify factors associated with EV-D68 seropositivity. After adjusting for age group, sex, and ARTI status, no variable emerged as a statistically significant independent predictor (Table 4). The adjusted odds ratio (aOR) for ARTI was 1.42 (95% CI: 0.46–4.41, *p* = 0.542), indicating that respiratory symptoms were not independently associated with seropositivity after controlling for age.

Since seropositivity was near-saturated (96.0%), we additionally evaluated factors associated with log_2_-transformed NtAb titers using ANCOVA, which provided greater statistical power. After adjusting for age and sex, ARTI status was associated with a marginally higher GMT (adjusted mean difference in log_2_ titer: 0.31, 95% CI: −0.02 to 0.64, *p* = 0.067), consistent with the borderline distribution difference observed in Table 2.

Given that five predictors were tested simultaneously in this model, we applied the Benjamini–Hochberg procedure to control the false discovery rate. After correction, no predictor remained statistically significant, and the results should therefore be interpreted as exploratory.

### 3.6. Nonparametric Sensitivity Analyses

Given the wide range of NtAb titers (<1:16 to >1:1024), we performed nonparametric sensitivity analyses to verify the robustness of the parametric findings (Table 5). The Shapiro–Wilk test confirmed that log_2_-transformed titers significantly deviated from normality (W = 0.9354, *p* < 0.001), validating the use of nonparametric methods. The Mann–Whitney U test revealed that NtAb titers were significantly higher in ARTI children compared to non-ARTI children (U = 29,726, Z = 2.96, *p* = 0.003), consistent with—and more sensitive than—the ANCOVA result (*p* = 0.067). This difference likely reflects the greater robustness of the nonparametric test to the bounded, discrete titer distribution. The Kruskal–Wallis test showed no significant difference in titer distributions across the four age groups (H = 4.59, df = 3, *p* = 0.205), consistent with the age-stratified ANCOVA, with pairwise age-group comparisons detailed in Table 6. Spearman’s rank correlation between age and log_2_ titer was weakly positive but did not reach statistical significance (ρ = 0.086, *p* = 0.067), consistent with the non-monotonic GMT trajectory observed in Figure 2. Overall, the nonparametric analyses confirmed and, in the case of the ARTI comparison, strengthened the conclusions drawn from the parametric approach.

## 4. Discussion

This cross-sectional serological survey in children attending Xiamen Children’s Hospital, conducted in 2022, revealed an overall EV-D68 neutralizing antibody seroprevalence of 96.0%, with rates exceeding 90.0% in every age group and reaching 100% in infants under one year. This remarkably high prevalence, comparable to the near-universal levels typically observed only in adult populations [13], indicates that EV-D68 has been circulating endemically and extensively among children in this region, establishing a high population immune baseline. Globally, reported EV-D68 seroprevalence rates are generally high, with most estimates ranging from 70% to over 90%, despite inter-study differences in age ranges, assay methods, and seropositivity thresholds (1:4–1:16) [7,8,9,10,11,12,13,14,15,16,21]. From a seroepidemiological perspective, such data are invaluable: they quantify the cumulative infection burden, delineate age-specific susceptibility windows, and provide a reference against which future changes in transmission intensity or antigenic evolution can be measured.

To address the potential violation of normality given the wide titer range, we additionally performed nonparametric sensitivity analyses. The Shapiro–Wilk test confirmed significant non-normality of log_2_-transformed titers (*p* < 0.001). The Mann–Whitney U test detected a nominally significant difference between ARTI and non-ARTI groups in this nonparametric framework (*p* = 0.003) that was consistent in direction with the ANCOVA trend (*p* = 0.067) but achieved greater statistical power, likely because the nonparametric approach is better suited to the bounded, discrete titer distribution. The Kruskal–Wallis test (*p* = 0.205) and Spearman correlation (ρ = 0.086, *p* = 0.067) corroborated the absence of a strong monotonic age effect, consistent with the non-linear GMT trajectory. These nonparametric results strengthen the credibility of our findings.

Among hospital-attended children, our estimate is substantially higher than the global pooled seroprevalence of 76.0% reported in a recent meta-analysis of studies published before 2022 [21]. It also exceeds the pre-pandemic figures from several other regions, where seropositivity typically climbs gradually through childhood, reaching ~50.0% by age 2 and approaching 100% only in adulthood [7,10,12,13]. Notably, the universal seropositivity in infants < 1 year is striking. Although EV-D68-specific PCR testing was not performed in our study cohort, molecular surveillance data from the same region provide complementary evidence of active virus circulation. A recent multicenter study screening 2842 children with community-acquired pneumonia across five eastern Chinese provinces, including Fujian, during 2021–2024 detected EV-D68 in 0.7% of cases, with a biennial peak pattern (1.2% in 2022, 1.1% in 2024) [22]. Subclades B3 and D3 co-circulated, with D3 emerging as the dominant lineage in severe pneumonia cases in 2024. These molecular findings corroborate the high seroprevalence observed in our study and confirm that EV-D68 continues to circulate actively in eastern China, including Fujian province. While maternal antibodies can persist for several months, they generally wane by 6 months of age [23]. For infants aged < 6 months, the presence of NtAbs likely reflects residual maternal immunity, whereas for those aged 6–12 months, the high GMT and the presence of titers ≥ 1:512 in some cases are more consistent with active infection acquired through intra-familial transmission. This inference is supported by household transmission studies that have documented secondary attack rates of 15–31.0% among contacts and prolonged viral shedding [24,25]. Our serological finding thus highlights the role of household networks as a key driver of early childhood infection, a pattern that serosurveillance can help to elucidate.

The age-specific antibody profiles revealed three distinct immunological phases, which are of central importance for risk assessment. First, infants under 1 year had the highest GMT (86.31) and the largest proportion of high-titer responses, probably reflecting a combination of residual maternal antibody protection. The 100% seropositivity in this age group, combined with the high GMT, suggests that transplacental IgG transfer from mothers with high EV-D68 antibody titers—themselves likely exposed during the intense post-pandemic transmission period—confers robust passive immunity during the first months of life. This has direct translational implications: maternal vaccination or boosting strategies could potentially extend the duration of protection through the vulnerable toddler window. Second, children aged 1–3 years exhibited the lowest GMT (63.52) and the highest proportion of low-titer (1:16–1:32) sera (44.7%), representing a clear “immune gap” after maternal antibodies have faded and before the child’s adaptive system has been sufficiently boosted by repeated exposures. This age window is epidemiologically critical, as it may correspond to increased susceptibility to both infection and severe outcomes, including AFM [13]. Third, school-aged children (≥5 years) displayed stable mid-range titers and the greatest frequency of high-titer responses (≥1:64: 64.1%), indicative of cumulative boosting through repeated viral encounters that consolidate humoral memory. The lowest seroprevalence (92.6%) was observed in the 3–5-year group, particularly among non-ARTI children (89.8%), reinforcing the concept of a toddler susceptibility window that should be prioritized in surveillance and, should interventions become available, in targeted strategies.

Although seroprevalence did not differ between ARTI and non-ARTI children, we consistently observed higher antibody titers in the ARTI group: a nominally lower proportion of low-titer sera (28.6% vs. 39.0%; *p* = 0.025) and a borderline global distribution difference (*p* = 0.053). The adjusted ANCOVA further estimated that ARTI children had a GMT approximately 19.0% higher than non-ARTI children (84.2 vs. 70.5), although this difference did not reach statistical significance (*p* = 0.067). However, the interpretation of this finding is fundamentally constrained by the absence of EV-D68-specific molecular testing in the ARTI group. Several alternative explanations must be considered. First, the higher titers may reflect recent or concurrent EV-D68 infection genuinely contributing to the respiratory symptoms—a hypothesis that cannot be tested without PCR confirmation. Second, they may represent pre-existing high baseline immunity from repeated prior exposures that is incidental to the current illness episode, particularly given that 96.0% of all children were seropositive. Third, cross-reactive anamnestic boosting by other respiratory pathogens (e.g., rhinovirus, other enteroviruses) during the acute illness could elevate EV-D68 antibody titers non-specifically [26,27]. Fourth, the ARTI group was significantly younger than the non-ARTI group (median 3.0 vs. 5.0 years, *p* < 0.001), and while we adjusted for age in all multivariable models, residual confounding cannot be entirely excluded. It should also be noted that among the five individual titer stratum comparisons in Table 2, four yielded *p* > 0.05, and the single nominally significant comparison (1:16–1:32 stratum, nominal *p* = 0.025) did not survive Bonferroni correction (adjusted *p* = 0.125). The global χ^2^ test for the overall titer distribution (*p* = 0.053, not statistically significant) similarly fell short of the conventional significance threshold. These statistical observations further reinforce our conclusion that the ARTI–non-ARTI titer comparison should be regarded as exploratory. For these reasons, the ARTI–non-ARTI comparison should be regarded strictly as exploratory and hypothesis-generating. The descriptive age-stratified seroprevalence estimates and GMT trajectories, which constitute the primary objectives of this study, are unaffected by these limitations.

The near-saturation of seropositivity (96.0%) implies that a large proportion of this hospital-attended pediatric cohort has been primed, which may dampen the short-term risk of large outbreaks in hospital-attended pediatric populations. However, seroepidemiology does not merely measure past exposure; it also serves as a sentinel for changing viral dynamics. The ongoing evolution of EV-D68-with the emergence of B3 and D3 subclades in China since 2021 underscores the need for continuous serological monitoring, as genetic drift could alter antigenicity and reduce the protective efficacy of existing immunity. Our age-stratified GMT data provide a quantitative baseline against which future serosurveys can be compared to detect waning immunity or antigenic mismatch. Such comparisons are essential for outbreak forecasting and for evaluating the potential need for targeted interventions in susceptible age groups.

The high seroprevalence observed in this study also has implications for the risk of severe neurological outcomes. EV-D68 is strongly associated with acute flaccid myelitis (AFM), a rare but devastating complication predominantly affecting children. AFM outbreaks in China have been documented, including an 18-case cluster in Zhejiang province in 2018 in which 50% of cases tested positive for EV-D68 subclade B3 [28], and the first molecularly confirmed EV-D68-associated AFM case in Beijing [29]. Although AFM case data were not systematically collected in the present serological survey, the near-universal seropositivity (96.0%) suggests that the pediatric population in Xiamen has been broadly exposed to EV-D68. This high immune baseline may confer partial protection against severe neuroinvasive disease upon reinfection, consistent with the fact that AFM remains rare even in settings with high EV-D68 seroprevalence. However, continued vigilance is warranted, as the ongoing evolution of subclades B3 and D3—both of which have been associated with severe respiratory disease in eastern China [22]—could alter antigenic profiles and potentially increase the risk of breakthrough neurological complications. We recommend that future serosurveys in this region be paired with active AFM case ascertainment and EV-D68-specific molecular testing to enable direct correlation of serological status with clinical outcomes.

Beyond clinical and serological surveillance, environmental monitoring through wastewater-based epidemiology has emerged as a valuable complementary approach for tracking enterovirus circulation. Recent studies have demonstrated that EV-D68 RNA can be detected in sewage, with wastewater concentrations temporally correlated with clinical case surges in the United States and Europe. In China, the first detection of EV-D68 in sewage was recently reported in Jinan in 2024, where next-generation sequencing of wastewater samples revealed co-circulation of multiple enterovirus types including D68 [30]. However, no environmental surveillance program for EV-D68 currently exists in Xiamen or Fujian province.

Cross-regional comparison with other Chinese pediatric serosurveys further contextualizes our findings: Liu et al. (2021) reported 77.3% seroprevalence in Beijing children aged 0–15 years (2012–2017) [14], while Sun et al. (2018) documented 85.2% in children aged 1–5 years across multiple Chinese provinces (2012–2016) [16]. Our 96.0% estimate in 2022 exceeds all previously reported Chinese pediatric seroprevalence figures, highlighting the exceptional transmission intensity in this coastal city during the post-pandemic period and underscoring the value of this dataset as a regional serological benchmark.

Given the 96.0% seroprevalence documented in this study, which implies extensive community-level viral exposure, the establishment of wastewater monitoring for EV-D68 in this region would provide a valuable, cost-effective sentinel system for early detection of circulation peaks and emerging subclades. We recommend that future studies integrate environmental surveillance with clinical and serological monitoring to achieve comprehensive, multi-modal assessment of EV-D68 epidemiology.

Several limitations should be acknowledged. The hospital-based design may introduce selection bias, limiting generalizability to the general pediatric population. The use of a single reference strain (STL, clade B) may not fully represent the neutralizing activity against circulating clades B3 and D3. The absence of paired acute-convalescent sera precludes confirmation of recent infection, and maternal antibody contributions in infants cannot be definitively separated from active infection. Furthermore, key epidemiological covariates known to influence enterovirus exposure—including daycare attendance, household size (number of children), and recent respiratory virus exposure history—were not systematically documented in the electronic medical records at Xiamen Children’s Hospital during the study period and could not be incorporated into our regression models. The absence of these variables limits the analytical depth of our antibody-associated factor analysis; previous EV-D68 serosurveys that incorporated daycare attendance and household crowding have demonstrated significant associations between these factors and seropositivity in young children [8,10]. The adjustment for only age and sex in our ANCOVA and Firth regression models may therefore leave residual confounding from unmeasured environmental exposure variables, and our antibody titer associations should be interpreted with this limitation in mind. Additionally, cross-reactivity with other enteroviruses may have led to overestimation of seroprevalence. However, poliovirus was not included in the cross-reactivity panel, and more importantly, even with a specific detection antibody, polyclonal serum antibodies from study participants may contain cross-reactive antibodies elicited by prior infections with other enteroviruses that share antigenic epitopes with EV-D68 [26]. Such cross-reactive antibodies could potentially influence the neutralization readout, leading to overestimation of true EV-D68-specific titers. Nonetheless, several considerations argue against a major assay-specific bias: (i) the age-stratified seroprevalence pattern is consistent with known EV-D68 epidemiology and differs from the typical seroepidemiology of EV-A71 or coxsackieviruses; (ii) neutralization assays are inherently more specific than binding assays because they require functional virus–antibody interaction. Furthermore, the ARTI group was defined syndromically without standardized operational criteria for temperature thresholds, symptom duration, or radiographic confirmation, which may have introduced heterogeneity in case ascertainment. Most importantly, the absence of EV-D68-specific PCR testing in the ARTI group precludes any etiologic attribution of respiratory symptoms to EV-D68 infection. The ARTI–non-ARTI comparison is therefore presented as an exploratory analysis, and its findings should not be interpreted as evidence of EV-D68-associated respiratory disease. Future community-based, longitudinal serosurveys, ideally combined with molecular typing, are needed to validate and extend our findings.

Furthermore, this study involved approximately 39 hypothesis tests across demographic comparisons, titer distribution analyses, regression models, and sensitivity analyses. Although we have now applied Bonferroni or Benjamini–Hochberg corrections to the most critical families of tests (titer stratum comparisons, Firth regression predictors, and pairwise age-group contrasts), the overall multiplicity of testing warrants caution. In particular, the nominal *p* = 0.025 for the 1:16–1:32 titer stratum in Table 2 and the global χ^2^ *p* = 0.053 should be regarded as exploratory findings that require independent replication rather than confirmatory evidence. We encourage future studies with larger, prospectively designed cohorts to validate these observations using pre-specified primary endpoints to minimize multiplicity concerns.

## 5. Conclusions

This cross-sectional hospital-based serological survey of 453 children aged 0–16 in Xiamen, China, after COVID-19 control measures yielded a high overall EV-D68 neutralizing antibody seroprevalence of 96.0%, with seropositivity exceeding 90% across all age strata, which proves persistent endemic circulation of EV-D68 in this hospital-attended pediatric population despite pandemic transmission interruptions. A distinct immune vulnerability window exists in toddlers aged 1–3 years, who carried the lowest geometric mean antibody titers and the largest proportion of low-level neutralizing responses; this age group should be prioritized for clinical surveillance of acute AFM and severe respiratory complications associated with EV-D68. The exploratory comparison between children with and without ARTI revealed nominally higher antibody titers in the symptomatic group; however, the absence of EV-D68-specific PCR testing precludes any causal inference regarding the etiologic role of EV-D68 in these respiratory episodes, and this analysis should be regarded as hypothesis-generating only. For future public health and virological research, long-term community-based longitudinal serological cohorts combined with routine viral whole-genome sequencing are strongly recommended to track antibody waning dynamics, monitor antigenic shifts in circulating EV-D68 subclades, and precisely quantify age-specific outbreak risks in pediatric populations.

## Figures and Tables

**Table 1:** Demographic and clinical characteristics of the 453 enrolled children, stratified by ARTI status.

Characteristic	Total (*n* = 453)	With ARTI (*n* = 217)	Without ARTI (*n* = 236)	Statistic	*p*
Mean age (IQR), years	4.0 (2.0–7.0)	3.0 (1.0–6.0)	5.0 (3.0–9.0)	*U* = 17,646	<0.001
Age subgroups, *n* (%)					
0–1 year	51 (11.3)	33 (15.2)	18 (7.6)	*X*^2^ = 5.765	0.016
1–3 y	85 (18.8)	51 (23.5)	34 (14.4)	*X*^2^ = 5.553	0.018
3–5 y	108 (23.8)	59 (27.2)	49 (20.8)	*X*^2^ = 2.229	0.135
≥5 y	209 (46.1)	74 (34.1)	135 (57.2)	*X*^2^ = 23.358	<0.001
Male *n* (%)	278 (61.4)	134 (61.8)	144 (61.0)	*X*^2^ = 0.004	0.949
Overall seroprevalence (≥1:16), *n* (%)	435 (96.0)	210 (96.8)	225 (95.3)	*X*^2^ = 0.292	0.589

Continuous variables are presented as median (interquartile range) and compared using the Mann–Whitney *U* test. Categorical variables are presented as *n* (%) and compared using Pearson’s χ^2^ test. The “Statistic” column reports the test statistic for the ARTI vs. non-ARTI comparison. ARTI: acute respiratory tract infection.

**Table 2:** Distribution of EV-D68 neutralizing antibody titers in the overall cohort and by ARTI status.

Titer Stratum	Total (*n* = 453)	With ARTI (*n* = 217)	Without ARTI (*n* = 236)	*X* ^2^	*p*
<1:16 (seronegative)	18 (4.0%)	7 (3.2%)	11 (4.7%)	0.292	0.589
1:16–1:32	154 (34.0%)	62 (28.6%)	92 (39.0%)	5.007	0.025
1:64–1:256	218 (48.1%)	113 (52.1%)	105 (44.5%)	0.005	0.942
1:512–1:1024	27 (6.0%)	12(5.5%)	15 (6.4%)	2.016	0.156
>1:1024	36 (7.9%)	23 (10.6%)	13 (5.5%)	3.339	0.068
Global distribution	-	-	-	9.357 (df = 4)	0.053

Nominal *p* values from χ^2^ tests for individual titer strata are presented. After Bonferroni correction for five comparisons (adjusted α = 0.010), none of the individual stratum comparisons reached statistical significance.

**Table 3:** Age- and ARTI-stratified seroprevalence and key titer metrics.

Age Group	All (*n* = 453)		With ARTI (*n* = 217)		Without ARTI (*n* = 236)	
	Seropositive, %	GMT	Seropositive, %	GMT	Seropositive, %	GMT
0–1 y	100	86.31	100	90.15	100	78.52
1–3 y	97.6	63.52	98.0	68.24	97.1	57.83
3–5 y	92.6	69.14	94.9	74.56	89.8	62.41
≥5 y	96.2	80.47	95.9	83.92	96.3	78.11

Nominal *p* values are presented without adjustment for multiple comparisons and should be interpreted with appropriate caution.

**Table 4:** Multivariable Firth’s penalized logistic regression for factors associated with EV-D68 seropositivity (titer ≥ 1:16).

Variable	aOR	95% CI	*p*
Age group (ref: 0–1 y)			
1–3 y	0.42	0.05–2.61	0.368
3–5 y	0.18	0.02–1.04	0.064
≥5 y	0.31	0.04–1.73	0.194
Sex (ref: female)	0.89	0.32–2.56	0.834
ARTI (ref: no)	1.42	0.46–4.41	0.542

**Table 5:** Nonparametric sensitivity analyses for NtAb titer comparisons.

Analysis	Test	Statistic	*p* Value
Normality of log_2_ titers	Shapiro–Wilk	W = 0.9354	<0.001
ARTI vs. non-ARTI (titers)	Mann–Whitney U	U = 29,726; Z = 2.96	0.003
Age groups (titers)	Kruskal–Wallis	H = 4.59, df = 3	0.205
Age (continuous) vs. titer	Spearman ρ	ρ = 0.086	0.067

**Table 6:** Pairwise Mann–Whitney U between age groups (Bonferroni-adjusted).

Comparison	U	*p* (Raw)	*p* (Adjusted)
0–1 y vs. 1–3 y	2572	0.064	0.383
0–1 y vs. 3–5 y	3037	0.29	1
0–1 y vs. ≥5 y	5380	0.915	1
1–3 y vs. 3–5 y	4366	0.556	1
1–3 y vs. ≥5 y	7689	0.067	0.404
3–5 y vs. ≥5 y	10,434	0.265	1

## Data Availability

The data presented in this study are available upon reasonable request from the corresponding authors.
